# Perceptual expertise improves category detection in natural scenes

**DOI:** 10.3758/s13423-015-0872-x

**Published:** 2015-06-24

**Authors:** Reshanne R. Reeder, Timo Stein, Marius V. Peelen

**Affiliations:** Center for Mind/Brain Sciences, University of Trento, 38068 Rovereto, Italy; Institute for Psychology, Otto von Guericke University, 39106 Magdeburg, Germany

**Keywords:** Visual search, Within-category identification, Discrimination, Object recognition

## Abstract

There is much debate about how detection, categorization, and within-category identification relate to one another during object recognition. Whether these tasks rely on partially shared perceptual mechanisms may be determined by testing whether training on one of these tasks facilitates performance on another. In the present study we asked whether expertise in discriminating objects improves the detection of these objects in naturalistic scenes. Self-proclaimed car experts (*N* = 34) performed a car discrimination task to establish their level of expertise, followed by a visual search task where they were asked to detect cars and people in hundreds of photographs of natural scenes. Results revealed that expertise in discriminating cars was strongly correlated with car detection accuracy. This effect was specific to objects of expertise, as there was no influence of car expertise on person detection. These results indicate a close link between object discrimination and object detection performance, which we interpret as reflecting partially shared perceptual mechanisms and neural representations underlying these tasks: the increased sensitivity of the visual system for objects of expertise – as a result of extensive discrimination training – may benefit both the discrimination and the detection of these objects. Alternative interpretations are also discussed.

Detection (“is there an object?”), categorization (“is the object a car?”), and within-category identification (“is the car a Honda Civic?”) are all part of object recognition, but there is much debate about how these processes relate to one another (Delorme, Rousselet, Macé, & Fabre-Thorpe, 2004; Grill-Spector & Kanwisher, 2005; Large, Kiss, & McMullen, 2004; Mack, Gauthier, Sadr, & Palmeri, 2008; Rosch, Mervis, Gray, Johnson, & Boyes-Braem, 1976; Thorpe, Fize, & Marlot, 1996). Detection and categorization can be similarly efficient, whereas within-category identification is typically a much slower process (Grill-Spector & Kanwisher, 2005), suggesting that identification involves additional perceptual mechanisms. For example, categorizing an object as a car is presumably achieved through the recognition of visual features that are diagnostic for cars: that is, features that are shared by most cars but not other object categories (e.g., the shape of the rim/wheels; Harel, Ullman, Harari, & Bentin, 2011; Reeder & Peelen, 2013; Ullman, 2007; Ullman, Vidal-Naquet, & Sali, 2002). Conversely, discriminating among specific exemplars within a particular category relies on more fine-grained visual information (Collin & McMullen, 2005) and on specific spatial configurations of object parts, which requires a slower, more detailed level of processing relative to simple detection and categorization (Grill-Spector & Kanwisher, 2005; Op de Beeck & Baker, 2010).

Although different features are diagnostic for object categorization and within-category identification, there is some evidence that identification-related perceptual mechanisms affect object categorization. In particular, perceptual expertise in discriminating between exemplars of a specific, homogeneous object category (e.g., cars) can influence performance in tasks that do not require within-category discrimination. For example, people who are experts at distinguishing individual exemplars of cars are faster than novices at categorizing image fragments of cars, indicating that perceptual expertise may entail a more general processing advantage for objects from the category of expertise (Harel et al., 2011). Previous studies of car experts have also found that presenting cars as irrelevant distractors during search for faces interferes with face detection, suggesting that extensive individuation training on an object category can influence the extent to which those objects recruit perceptual mechanisms involved in the processing of a natural category of expertise (McGugin, McKeeff, Tong, & Gauthier, 2011; McKeeff, McGugin, Tong, & Gauthier, 2010). Indeed, there is neuroimaging evidence that the perception of objects of expertise (e.g., faces and cars for car experts) involves cortical regions distinct from those representing other objects (e.g., McGugin, Gatenby, Gore, & Gauthier, 2012), and such specialized neural processing may benefit even simple category detection. In a study by Hershler and Hochstein (2009), bird and car experts searched through visual arrays of various distractor objects for a single photograph of a bird, car, or face in separate blocks of trials. Results revealed faster and more accurate detection of objects from the expert category compared to the non-expert category. Although there was only a small number of experts in this experiment (five car experts and six bird experts), the finding that within-category identification ability can have such an impact on detection and categorization suggests that these tasks may rely on partially shared perceptual mechanisms and neural representations.

To gain further insight into the effect of within-category identification ability on simple categorization and detection, in the current study we investigated the influence of perceptual expertise on category detection in natural scenes. Our study was designed to provide additional evidence for the impact of perceptual expertise on category detection and to explore whether this influence extends to real-world stimuli. Previous evidence for the beneficial effect of perceptual expertise on category detection was obtained by testing a small number of experts with each group acting as a novice control group for the other (Hershler & Hochstein, 2009). It is therefore possible that selection bias may have played a role in shaping the observed effects; that is, car experts may have found experimental blocks of the car detection task more interesting than blocks of the bird detection task (and vice versa for bird experts), leading to significant performance differences that were due to non-specific differences in motivation and vigilance rather than to perceptual expertise per se. To circumvent these concerns, we tested a large group of self-proclaimed car experts and correlated their degree of objectively assessed car discrimination expertise with performance on a visual search task that involved detecting cars in natural scene photographs. If perceptual expertise is associated with improved category detection, we expected car expertise to be correlated with car detection performance. This approach obviates the need for a novice control group, thereby greatly reducing the possibility that results could be contaminated by selection biases.

Whereas previous work used artificial visual search arrays with one fixed target photograph per category that was clearly separated from other objects in the display (Hershler & Hochstein, 2009), in the present study car experts were cued to detect people or cars in a large set of diverse real-world photographs in which targets could appear in various positions, sizes, and levels of occlusion. We hypothesized that if the perceptual mechanisms supporting discrimination of individual exemplars and those guiding category detection rely on partially shared object representations, car experts with relatively greater car discrimination ability should also show relatively more efficient detection of cars in natural scenes.

## Methods

### Participants

Thirty-four self-proclaimed car experts (two women) participated in the current study for payment. Participants were between the ages of 19 and 63 years (mean age = 26.0 years) and had normal or corrected-to-normal vision. Four participants were left-handed. Participants were recruited from the Rovereto and Trento communities by responding to fliers that called for car experts to participate in the experiment. Thirty participants had completed at least some university education, and the other four had completed a high school education.

### Stimuli

All stimuli were presented on a 19-inch Dell 1905 FP monitor with a screen resolution of 1280 × 1024 pixels and 60 Hz refresh frequency (Dell Inc., Round Rock, TX). Observers sat 57 cm from the screen. Stimuli were presented using “A Simple Framework” (Schwarzbach, 2011), a toolbox based on the Psychophysics Toolbox for MATLAB (MathWorks, Inc., Natick, MA, USA).

**Fig. 1:**
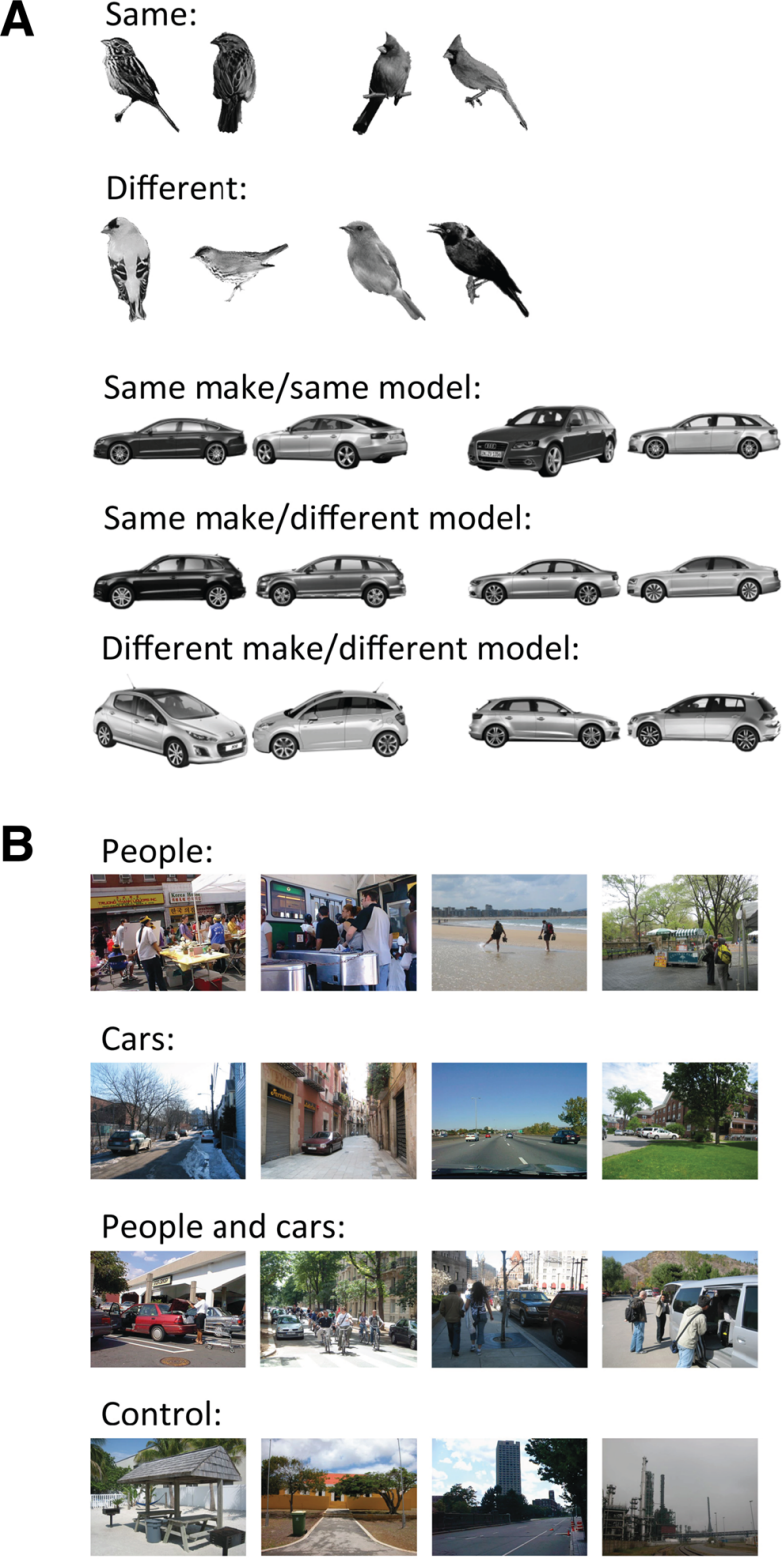
**a** Examples of the stimulus pairs used in the discrimination tasks of the expertise assessments and **b** examples of the natural scene photographs in the category detection task

Stimuli in the expertise assessments were 320 centrally presented black and white photographs of cars (160) and birds (160) shown in isolation on a white background. Images of modern cars (no more than ~5 years out of production) commonly seen on European streets were retrieved from free-access online image searches. A car expert created pairs of cars that were difficult to distinguish as belonging to the same or different make or model based on perceptual similarities alone. We additionally ensured that names written on the side of the car or on the license plate were not visible in the selected images. A logo was visible in some images, but we ensured that logos were not visible on both cars in a pair. Forty pairs of cars were the same make and model, 20 were the same make but different models, and 20 were different makes and models, for a total of 80 pairs of cars. Cars of even the same make and model could appear in different positions and colors, and could be from different years and series. Bird images were the same as those used by Gauthier and colleagues in previous experiments (e.g., Gauthier, Skudlarski, Gore, & Anderson, 2000), with 40 pairs belonging to the same species and 40 pairs belonging to different species. See Fig. 1a for some examples of stimuli in the expertise assessments.

Images of cars were fit inside a 300 × 300 pixel-sized box (by scaling the longer axis and adjusting the shorter axis proportionally) and images of birds were scaled to fit inside a 256 × 256 pixel-sized box, subtending 8.8° and 7.5° of visual angle, respectively. The size of the bird images was not changed from their original source and was adequate for identification as determined by a bird expert, whereas the size of the car images was adjusted to be adequate for identification as determined by a car expert.

A fixation cross and uppercased letter cues appeared centered on the screen in 70-point “bold” Times New Roman font. The fixation cross had dimensions of 31 × 31 pixels subtending 0.9° in height and width, and letters had dimensions of 70 × 70 pixels subtending 2.1° in height and width.

Stimuli presented in the category detection task were 960 color photographs (see Fig. 1b) of real-world scenes obtained from the LabelMe online database (Russell, Torralba, Murphy, & Freeman, 2008) and were divided into scenes containing cars (240), people (240), both cars and people (240), or neither cars nor people (240).

Scenes were scaled to 548 × 411 pixel resolution, subtending a visual angle of 16.1× 12.2°. Scenes were presented 7.4° from the center of the screen to the center of the image, above and below fixation.

### Experimental procedure

#### Expertise assessments

**Fig. 2:**
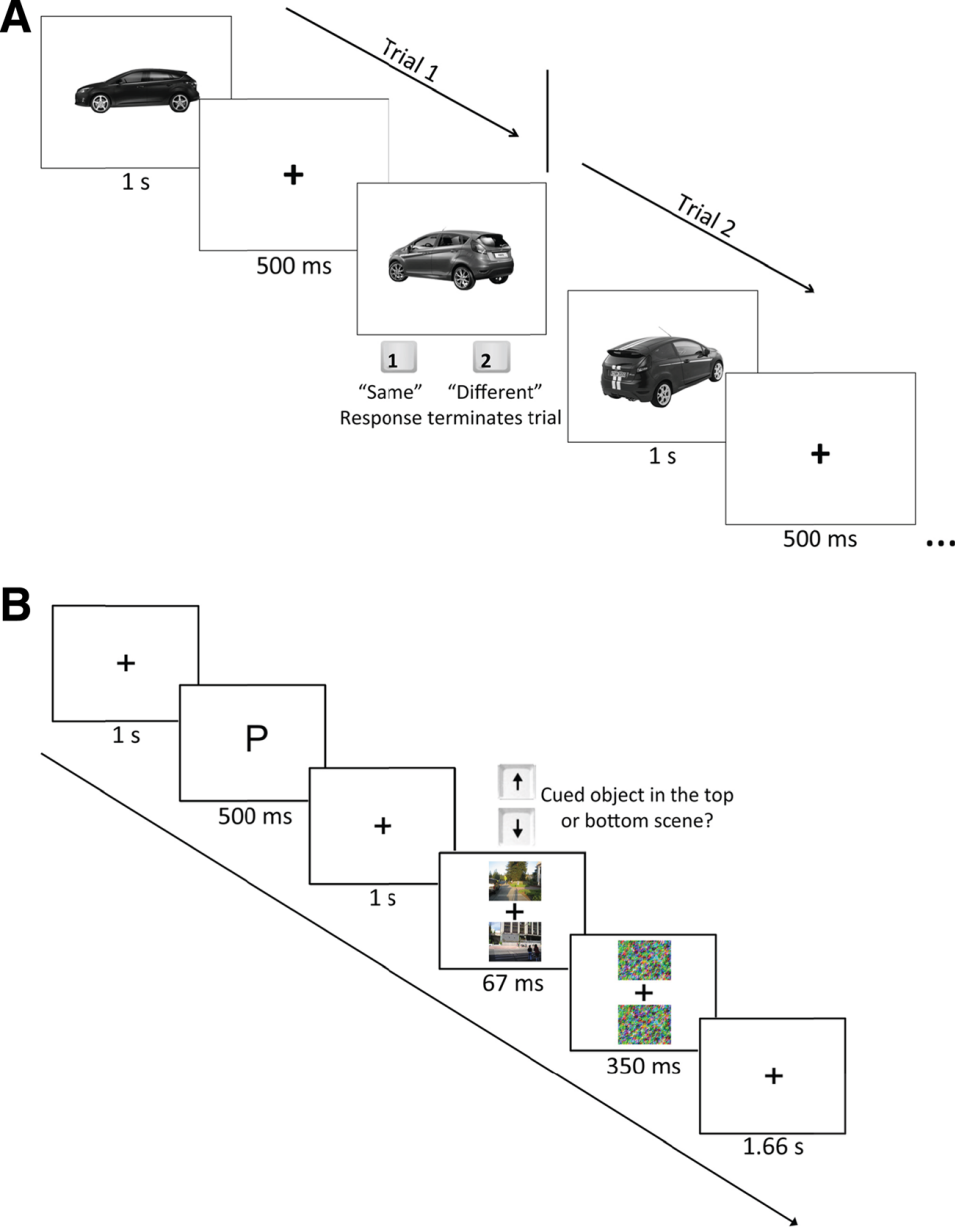
**a** The experimental paradigm of the discrimination tasks of the expertise assessments. Subjects completed one block each for cars (shown here) and birds. **b** The experimental paradigm of the category detection task

Prior to the category detection task, all participants completed a car and a bird discrimination task in separate blocks of 80 trials each (see Fig. 2a). In the car discrimination task, participants were required to determine whether two cars presented in succession were the same or different model (e.g, Honda Civic). On each trial, a car appeared in the center of the screen for 1 s, followed by a fixation for 500 ms, then another car that would remain on screen until the participant made a button press. Participants responded by pressing the “1” key on the number pad if they believed the two cars were the same model and the “2” key if they believed the two cars were different models. The same procedure was used for the bird discrimination task, except that two pictures of birds appeared instead of cars. Participants were required to respond whether they believed the two birds were the same (“1” key) or different (“2” key) species. Participants first completed the car block and then the bird block.

#### Category detection in natural scenes

All participants took part in one practice block followed by 8–10 blocks of 64 trials each. The category detection task started with the presentation of a fixation cross for 500 ms, followed by a single letter for 500 ms: “P” for “persona: or “M” for “macchina” (the Italian words for person and car, respectively). After the letter, another fixation cross appeared for 1 s, followed by two photographs of natural scenes for 67 ms which were immediately followed by a mask for 350 ms (Fig. 2b). Participants were required to respond whether the cued object category appeared above or below fixation using the “up” and “down” arrow keys, respectively. This spatial two-alternative forced-choice task was implemented to rule out response bias. The two scene photographs could either be one containing cars and the other containing people, or one containing both cars and people and the other containing no cars or people. This structure allowed us to present people and cars on every trial without making the location of one category informative of the location of the other. Each of the four scene types appeared in both possible locations an equal number of times. See Fig. 2b for a schematic representation of the category detection paradigm.

Within each experimental block, 16 attentional capture trials (see Reeder & Peelen, 2013) were randomly interspersed with the 64 category detection trials. These trials were not the focus of the current paper and results are not reported here.

### Analyses

The results section below reports correlations between expertise assessment performance and search performance. In the discrimination tasks, the average sensitivity score for cars (car *d’*) was 1.50 (SD = 1.21, range from −.34 to 4.74), and the average sensitivity score for birds (bird *d’*) was 1.18 (SD = .48, range from .39 to 1.95). These results are not directly comparable to previous assessments of expertise, as we used novel car stimuli that were more difficult to discriminate than those used in previous studies.

Correlations were computed between search performance and car *d’* (e.g., McGugin et al., 2012) as well as between search performance and car-bird *d’* (e.g., Curby & Gauthier, 2009; Gauthier et al., 2000). Both car *d’* and car-bird *d’* have been used as measures of expertise in previous studies. Whereas correlations between search performance and car *d’* provide directional information (i.e., positive correlations reflect better performance for higher scoring experts), correlations between search performance and car-bird *d’* alone are not informative of direction (i.e., positive correlations could either reflect a negative correlation with bird *d’* or a positive correlation with car *d’*) but provide a control for the possibility that correlations could be driven by general perceptual differences between participants.

## Results

**Fig. 3:**
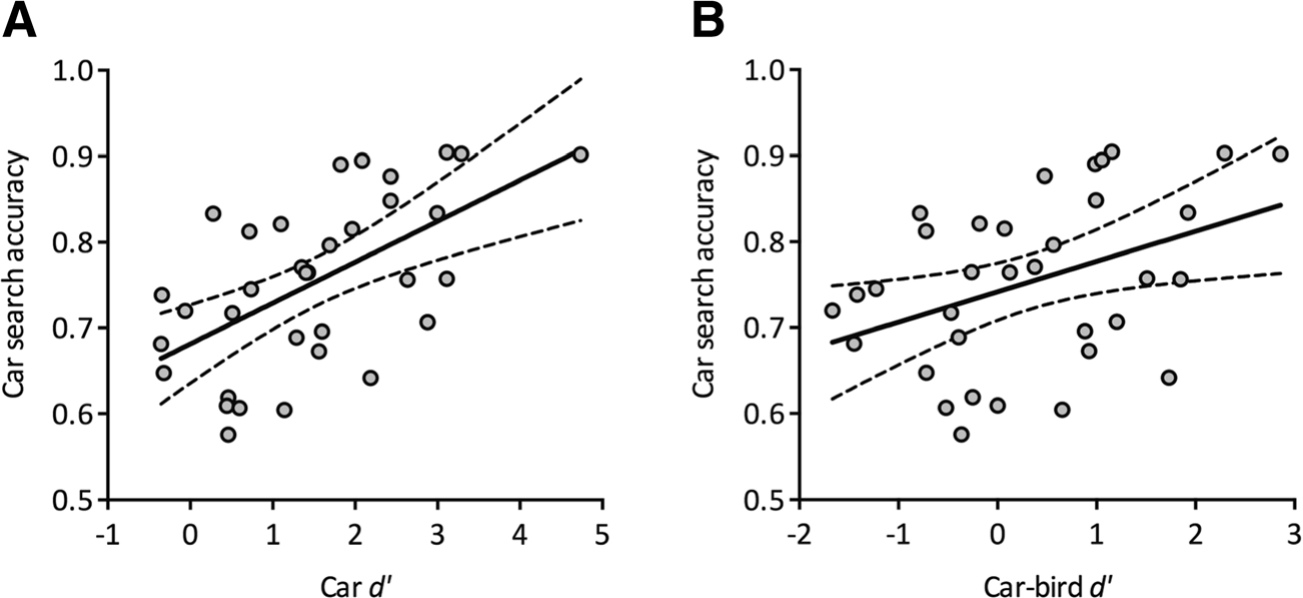
**a** The correlation between car *d’* and car search accuracy. **b** The correlation between car-bird *d’* and car search accuracy. Solid lines show the best-fitting linear regression lines and dashed lines show the 95 % confidence intervals

Sensitivity scores (*d’*) from the expertise assessments were correlated with accuracy from the category detection experiments using Spearman’s rank correlation (to minimize the influence of outliers). First, we directly analyzed the relationship between car discrimination expertise and car detection performance using car *d’* as the measure of expertise. As can be seen from Fig. 3a, there was a significant positive correlation between car *d’* and car detection accuracy, *r*(32) = .576, *p* < .001. There was no significant correlation between car *d’* and person detection accuracy, *r*(32) = .245, *p* = .163, but again a significant positive correlation between car *d’* and car−person accuracy, *r*(32) = .435, *p* = .010.

To control for the possibility that correlations with car *d’* alone were driven by generally better perceptual performance, we next correlated detection accuracy with another established measure of car expertise, namely the difference in *d’* scores between the car and the bird expertise assessment (car−bird *d’*). This analysis confirmed the previous results: there was a significant positive correlation between car−bird *d’* and car detection accuracy, *r*(32) = .399, *p* = .019 (Fig. 3b), no correlation between car−bird *d’* and person detection accuracy, *r*(32) = .118, *p* = .506, and a positive correlation between car−bird *d’* and car−person accuracy, *r*(32) = .401, *p* = .019.

There were no significant correlations between car *d’* or car−bird *d’* and response time (RT) for either car or person detection (all *p*s > .17). Furthermore, there was no significant correlation between person detection accuracy and person detection RT, *r*(32) = −.150, *p* = .397, although there was a small (non-significant) negative correlation between car detection accuracy and car detection RT, *r*(32) = −.266, *p* = .128, with shorter RTs for higher accuracies, indicating that these data were not affected by speed-accuracy tradeoff.

Given the large age range of our participants (ages 19–63 years), we tested correlations between performance and age, and further added age as a control variable in partial correlation analyses. Participant age was not significantly correlated with either car *d’* or car-bird *d’*, *r*s(32) < .195, *p*s > .26. Furthermore, age was not correlated with car detection accuracy or person detection accuracy, *r*s(32) < .081, *p*s > .65. We then conducted partial correlation analyses between expertise and detection accuracy with age as a control variable, and found significant correlations between car *d’* and car detection accuracy, *r*(31) = .596, *p* < .001, and between car-bird *d’* and car detection accuracy, *r*(31) = .412, *p* = .017. There were still no significant correlations between car *d’* or car-bird *d’* and person detection accuracy with age as a control variable, *r*s(31) < .273, *p*s > .12. Finally, the correlations between age and RTs in the car and person detection tasks were not significant (*r*(32) = .194, *p* = .271, and *r*(32) = .291, *p* = .094, respectively) .

## Discussion

The goal of the current study was to determine the effect of within-category identification ability on category detection in natural scenes. Specifically, we tested the effects of car discrimination expertise on car detection accuracy in a large set of real-world scene photographs. Results revealed that greater car expertise was associated with better car detection performance. Similar results were found when controlling for overall performance differences by subtracting bird expertise scores, and by subtracting person detection performance. These findings are consistent with a previous study by Hershler and Hochstein (2009) who found that experts, relative to novices, were better at detecting objects from their category of expertise in visual search arrays. Importantly, the present study used a correlation approach and extended these findings to more naturalistic viewing conditions, showing that the link between perceptual expertise and simple detection is unlikely to reflect motivational factors alone, and holds even for category detection in real-world scenes.

Our results are consistent with findings of superior detection ability for two other categories of objects that are frequently discriminated in everyday life: faces and bodies of people. Human faces and bodies are sometimes considered “natural categories of perceptual expertise,” and both human faces and bodies are detected more efficiently than other objects (Downing, Bray, Rogers, & Childs, 2004; Hershler, Golan, Bentin, & Hochstein, 2010; Hershler & Hochstein, 2005; Ro, Friggel, & Lavie, 2007; Simpson, Husband, Yee, Fullerton, & Jakobsen, 2014; Stein, Sterzer, & Peelen, 2012). Alternatively, however, enhanced face and body detection may reflect innate neural mechanisms (Johnson, 2005; Simion, Regolin, & Bulf, 2008). The current finding that car expertise improves car detection in natural scenes therefore provides more direct evidence that within-category identification expertise improves visual detection than that provided by previous studies on body and face perception.

The reason for adopting a correlation approach in the current study was to directly relate individual differences in car detection performance to individual differences in car discrimination expertise. By recruiting all participants similarly – appealing to their expertise and enthusiasm for cars – we aimed to reduce possible selection biases and potential motivational differences between relative experts and relative novices. Indeed, the absence of a significant correlation between car discrimination expertise and person detection performance rules out that the current results are due to general differences in motivation or vigilance. Nonetheless, we cannot exclude that observers with relatively greater car expertise were more motivated and attentive during car detection trials and that our findings partly reflect differences in such factors. Another possibility is that perceptual expertise necessarily entails more interest and attention for stimuli from the category of expertise (cf. Harel et al., 2011), and that expertise-specific effects may depend on the expert’s goals and degree of interest in a given task or situation (Harel, Kravitz, & Baker, 2013). For example, widespread increased visual cortex activity to objects of expertise is strongly reduced when these stimuli are ignored and interspersed with other objects (Harel, Gilaie-Dotan, Malach, & Bentin, 2010). More recent neuroimaging findings, however, suggest that focal expertise-related visual cortex activity is quite robust to manipulations of top-down attention (McGugin, Newton, Gore, & Gauthier, 2014: McGugin, Van Gulick, Tamber-Rosenau, Ross, & Gauthier, 2014).

The association between discrimination and detection performance is in line with studies on the neural basis of object recognition showing that the cortical regions recruited for these tasks overlap (Mason & Macrae, 2004; Tarr & Cheng, 2003). Extensive object discrimination training can lead to a reorganization of neural response profiles in inferotemporal cortex, such that more neurons become tuned to learned object exemplars (Logothetis, Pauls, & Poggio, 1995). Similar effects have also been observed with fMRI, showing category-selective responses to objects of expertise (Gauthier et al., 2000; McGugin et al., 2012). These selective representations, brought about by extensive experience, may facilitate both detection and discrimination processes. For example, category-selective areas in visual cortex have been shown to causally contribute to both discrimination (Pitcher et al., 2009) and detection (van Koningsbruggen, Peelen, & Downing, 2013) of objects of their preferred category (e.g., human bodies). Thus, object representations in high-level visual cortex may be strengthened by experience and training, facilitating both detection and discrimination of these objects.

An alternative, or additional, interpretation of the correlation between discrimination expertise and detection performance is that it reflects the experts’ training on both of these tasks. Although all participants in the current study were car enthusiasts, it is possible that the relatively strong car experts also more frequently searched for cars in their daily-life surroundings compared to the weaker car experts. Previous studies have shown that extensive visual search training leads to superior detection performance (Koller, Hardmeier, Michel, & Schwaninger, 2008; Manning, Ethell, Donovan, & Crawford, 2006; McCarley, Kramer, Wickens, Vidoni, & Boot, 2004; Nodine, Kundel, Lauver, & Toto, 1996). Future studies could rule out this alternative explanation by using controlled discrimination training regimes, such as in Wong, Palmeri, and Gauthier (2009). In that study, one group of subjects was given 10 hours of explicit individuation training on the novel object class “Ziggerins” while a second group was given 10 hours of categorization training on the same objects, thereby equating the degree to which objects were detected. The authors found that categorization training (in contrast to individuation training) was not sufficient to produce certain hallmarks of perceptual expertise (e.g., holistic processing; Gauthier & Tarr, 2002), which suggests a special role of individuation training in the development of perceptual expertise. In the context of the current study, we predict that the deeper perceptual learning induced by individuation training, relative to shallower training tasks that control for perceptual exposure, creates an added benefit in the detection of these objects of expertise.

To conclude, the current study tested whether perceptual expertise affects category detection in naturalistic scenes. Results showed that car discrimination expertise is correlated with car detection performance, thereby revealing a close link between these two tasks. We interpret this finding as reflecting partially shared perceptual mechanisms and neural representations underlying detection and discrimination tasks: the increased sensitivity of the visual system for objects of expertise -- as a result of extensive discrimination training -- may benefit both the discrimination and the detection of these objects. Future work using controlled discrimination training regimes is needed to provide further support for this interpretation.
